# Circulating MicroRNA in Breast Cancer

**DOI:** 10.3390/cancers18060900

**Published:** 2026-03-11

**Authors:** Alexander Sturzu, Ruixia Ma, Yaguang Xi

**Affiliations:** 1 Department of Pharmaceutical and Biomedical Sciences, College of Pharmacy, University of Georgia, Athens, GA 30602, USA; alexander.sturzu@uga.edu (A.S.);; 2 Innovations in Drug Discovery (IDD) Program, College of Pharmacy, University of Georgia, Athens, GA 30602, USA

**Keywords:** breast cancer, miRNA, circulating biomarkers, triple-negative breast cancer

## Abstract

**Background/Objectives:** Despite recent advances in breast cancer diagnostics, therapies and personalized medicine through genetic profiling, effective treatment of aggressive subtypes, particularly triple-negative breast cancer (TNBC), remains a considerable clinical challenge. MicroRNAs (miRNAs) are post-transcriptional regulators of gene expression that influence tumor progression and are detectable extracellularly in biofluids, where they are typically protected within extracellular vesicles (e.g., exosomes) or associated with RNA-binding proteins and lipoprotein complexes. This review integrates current evidence on oncogenic and tumor-suppressive extracellular miRNAs in breast cancer, with emphasis on subtype-specific functions and potential clinical relevance as liquid-biopsy biomarkers and therapeutic targets. **Methods:** A PubMed-based literature review (January 2000–February 2026) was conducted using search terms combining “breast cancer” with “miRNA/microRNA” and “circulating/plasma/serum/exosomal/extracellular vesicle.” Studies were prioritized if they provided validated targets/mechanisms and/or human clinical evidence for diagnostic, prognostic, or predictive utility; discrepant findings were evaluated in a subtype-aware framework. Findings were organized into functional categories (e.g., EMT/metastasis, cell-cycle/DNA damage, immune modulation, and hormone/growth factor signaling). Clinical and translational studies evaluating circulating miRNAs for diagnosis, prognosis, treatment response, and toxicity prediction were synthesized, together with key pre-analytical and analytical variables that affect reproducibility. **Results:** Across mechanistic and clinical studies, miR-21 and miR-155 recur as prominent oncogenic miRNAs, whereas miR-205 is frequently reported as a tumor-suppressive miRNA that is reduced in breast cancer and in circulation in several cohorts. Panels combining these miRNAs show promise for sensitive and specific breast cancer diagnostics. Additionally, several miRNAs show context- or subtype-dependent effects, with opposing activities reported between TNBC and estrogen receptor (ER)-positive disease (e.g., miR-17-92, miR-425, miR-181 family members, miR-31, and miR-24). **Conclusions:** Circulating miRNAs represent a promising class of minimally invasive biomarkers and potential therapeutic targets; however, translation is constrained by biological context dependence and by pre-analytical and analytical variability. Standardized protocols and rigorously validated, subtype-aware biomarker panels will be essential for clinical implementation and for enabling miRNA-informed precision oncology in breast cancer.

## Simple Summary

Breast cancer is the most common cancer type in women. Variability in hormone receptor expression creates different subtypes that vary in behavior, treatment response, and patient outcome. MicroRNA (miRNA) is a small molecule that regulates how genes are turned on and off in cells. Because of their small size, miRNAs can easily be released from the cells that generate them. Circulating miRNAs can influence cancer growth, spread, and immune interactions, but can also be analyzed as biomarkers for cancer diagnostics, prognosis and therapy guidance. This review summarizes how specific miRNAs contribute to breast cancer development, how their effects differ between tumor subtypes, and the clinical approaches to the use of miRNAs for diagnosis, prognosis, and therapy guidance.

## 1. Introduction

Breast cancer is the most commonly occurring cancer among women worldwide and one of the leading causes of cancer-related mortality [1]. Ongoing clinical research has resulted in significant advances in screening, diagnostics, and therapy [2]. However, challenges persist in early detection, prognostic stratification, and treatment personalization, particularly for aggressive or therapy-resistant subtypes [3]. Breast cancer is a biologically heterogeneous disease comprising multiple molecular subtypes with distinct clinical behaviors, therapeutic sensitivities, and prognostic outcomes [4].

Breast cancer classification and management rely heavily on receptor profiling [5]. The hormone receptors estrogen receptor (ER), progesterone receptor (PR), and human epidermal growth factor receptor 2 (HER2) are widely used biomarkers for diagnosis and therapeutic decision-making, and they also actively shape tumor biology [6,7].

- ER and PR are hormone receptors and their positivity is generally associated with favorable prognosis, slower disease progression, and a high likelihood of response to endocrine therapies such as tamoxifen or aromatase inhibitors [2,7,8]. However, late recurrences remain a clinical concern, necessitating prolonged follow-up strategies [9].
- HER2 overexpression or amplification, found in approximately 15–20% of cases, is historically linked to aggressive disease, higher recurrence rates, and worse survival [10]. More recently, HER2-targeted antibody–drug conjugates have expanded the benefit of HER2-directed therapy to patients with HER2-low metastatic breast cancer, with trastuzumab deruxtecan improving progression-free and overall survival compared with chemotherapy [11].
- Triple-negative breast cancer (TNBC) expresses none of the three receptors (ER, PR, and HER2) and accounts for approximately 15% of breast cancer cases. This type of cancer is associated with an aggressive clinical course, early recurrence, and poor prognosis [12]. Because TNBC lacks hormone receptors and HER2, chemotherapy was long the mainstay of systemic therapy until immune checkpoint inhibitors (ICIs) were recently approved in early-stage high-risk TNBC and in advanced settings [3,13,14].

ER, PR, and HER2 have become key determinants of clinical decision-making [5,10,13]. However, they do not fully capture the complexity of breast cancer biology. For example, analyses of neoadjuvant trial cohorts indicate that HER2-low tumors represent a biologically and clinically distinct subset, with characteristic molecular features and differential treatment response, underscoring the need for more refined biomarker stratification beyond standard receptor status [15]. A related area of interest is whether tumor receptor status can be inferred from circulating biomarkers.

### 1.1. MiRNAs and Their Relevance in Breast Cancer

MiRNAs are key post-transcriptional regulators that fine-tune gene expression between transcription and translation. They are small, non-coding RNAs that regulate gene expression at the post-transcriptional level, thereby influencing multiple cancer hallmarks, including proliferation, apoptosis, angiogenesis, and metastasis [16]. In breast cancer, dysregulated miRNA expression profiles have been associated with specific molecular subtypes, treatment resistance, and disease outcomes [17]. MiRNAs can be released into circulation via passive leakage from dying cells or active secretion in extracellular vesicles such as exosomes, making them highly stable and accessible in liquid biopsies [18]. Their potential to serve as diagnostic, prognostic, and predictive biomarkers makes miRNAs a potential target for translational research and future clinical integration [17].

### 1.2. Role of miRNAs as Circulating Biomarkers

Circulating biomarkers, detectable in blood or other body fluids, have emerged as a promising, minimally invasive complement to tissue-based diagnostics [19]. They enable real-time disease monitoring with low procedural risk and may facilitate early detection of recurrence or metastasis. In breast cancer, circulating tumor DNA (ctDNA), exosomal proteins, and miRNAs have attracted substantial interest due to their potential clinical utility [19]. In this review, we conducted a comprehensive literature search of the PubMed database to identify miRNAs relevant to breast cancer. MiRNAs consistently highlighted in recent review articles were prioritized. For each selected miRNA, we summarize the seminal studies that first described its role in breast cancer, as well as subsequent work reporting additional targets, pathways, and mechanistic insights. Finally, we discuss the emerging value of circulating miRNAs as biomarkers for diagnosis, prognostic assessment, and therapeutic decision-making, and we consider the prospects for miRNA-based therapeutics in breast cancer. We also highlight evidence that certain miRNAs can exhibit context-dependent, and sometimes opposing, effects in TNBC compared with ER-positive breast cancer.

## 2. Characteristics of miRNAs

### 2.1. Biogenesis and Function of miRNAs

MiRNAs regulate gene expression post-transcriptionally and thereby influence pathways involved in cellular metabolism and diverse cellular processes (e.g., proliferation, apoptosis, differentiation, and stress responses). These short non-coding nucleotide sequences of around 20–25 nucleotides are present in most eukaryotes, and their processing mechanism is highly conserved. The primary function of miRNA is post-transcriptional regulation of messenger-RNA (mRNA) [16]. After loading into Argonaute (AGO) proteins, mature miRNAs form the RNA-induced silencing complex (RISC), which commonly recognizes target sites in the 3′ untranslated region (UTR) through complementarity to the miRNA seed region (nucleotides 2–8). Within the RISC, miRNAs directly inhibit either initiation or elongation during mRNA translation but can also induce mRNA decay [16,20,21].

**Figure 1:**
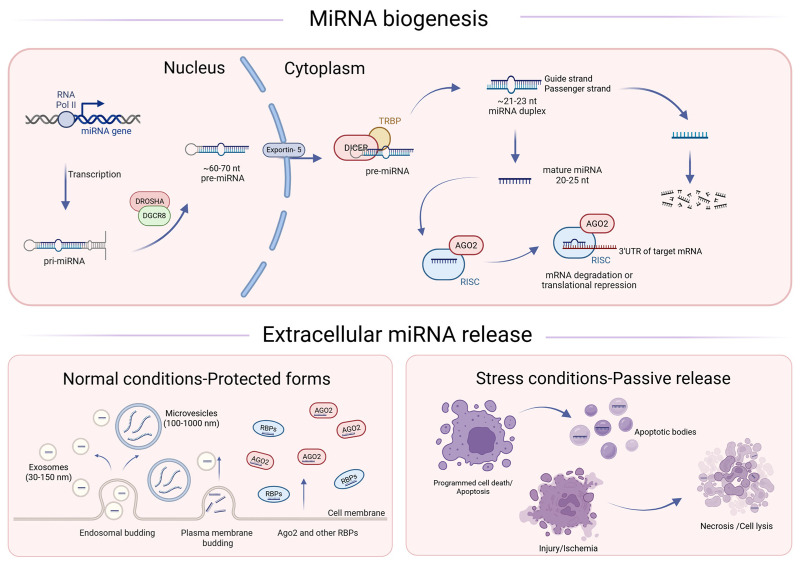
**Canonical miRNA biogenesis and extracellular release under normal and stress conditions.** MiRNA genes are transcribed by RNA polymerase II to generate primary miRNA transcripts (pri-miRNAs). In the nucleus, the microprocessor complex (DROSHA–DGCR8) cleaves pri-miRNAs into precursor miRNAs (pre-miRNAs), which are exported to the cytoplasm by Exportin-5 and processed by DICER in association with TRBP to produce a ~21–23 nt miRNA duplex. The guide strand is loaded into Argonaute (AGO2) to form the RNA-induced silencing complex (RISC), whereas the passenger strand is degraded. RISC-bound miRNAs recognize target sites in the 3′UTR of mRNAs to mediate translational repression and/or mRNA degradation. Under normal conditions, extracellular miRNAs are released predominantly in protected forms, including encapsulation within exosomes (endosomal origin) or microvesicles (plasma membrane shedding), and associated with AGO2 and other RNA-binding proteins (RBPs). Under stress or cell death, miRNAs can also be released in apoptotic bodies or passively during necrosis and cell lysis.

In animals, many miRNA genes are transcribed by RNA polymerase II to generate primary miRNA transcripts (pri-miRNAs) that contain one or more hairpin structures [22]. Pri-miRNAs are processed in the nucleus by the Microprocessor complex, which consists of the RNase III enzyme DROSHA and its essential double-stranded RNA-binding partner DGCR8 [23]. The resulting precursor miRNA (pre-miRNA) is exported to the cytoplasm by exportin-5 [24]. In the cytoplasm, the DICER ribonuclease cleaves the pre-miRNA hairpin to produce an ~22-nt miRNA duplex [22] (Figure 1). Because of the loop structure, this cleavage results in two mature miRNA sequences: the 5p sequence before the loop and a 3p sequence after the loop. The mature miRNAs form the RISC to effectuate their mRNA interactions [25,26]. An additional layer of complexity in miRNA biogenesis is the generation of miRNA isoforms (isomiRs), which can result from alternative DROSHA and DICER cleavage as well as post-processing modifications such as trimming or tailing [27,28]. Variations at the 5′ end can shift the seed region and thereby alter target recognition and regulatory output [28,29].

MiRNAs can also be detected extracellularly in biofluids. Rather than circulating as unprotected RNA, extracellular miRNAs are typically stabilized by association with Argonaute-containing ribonucleoprotein complexes, lipoproteins, or encapsulation within extracellular vesicles [30,31,32,33]. The following section discusses how these intracellular miRNAs translate into measurable extracellular biomarkers.

### 2.2. Mechanisms of miRNA Release and Stability

As miRNAs are very susceptible to degradation mediated by RNAses, the extracellular miRNAs are typically stabilized by association with carrier complexes, most commonly RNA-binding proteins and lipoproteins, or by encapsulation within membrane-bound extracellular vesicles.

Extracellular miRNAs can arise from both active secretion and passive release during cell injury or death. For example, apoptotic bodies (often reported across a broad size range, extending up to several micrometers) can contain miRNAs, and cellular lysis during necrosis may also contribute to extracellular miRNA pools, particularly in pathological settings such as cancer or ischemic injury [34]. One major form of extracellular miRNAs is found in ribonucleoprotein complexes, with AGO2 being a prominent carrier that protects miRNAs from degradation [34,35,36].

MiRNAs are also transported within extracellular vesicles. Exosomes, small vesicles of ~30–150 nm formed by endosomal budding of membranes and specific miRNAs, are actively packaged into them via RNA-binding proteins such as hnRNPA2B1, YBX1, and SYNCRIP [32,37,38]. Microvesicles are larger (~100–1000 nm) and bud off directly from the cell membrane [34]. In addition, extracellular miRNAs are found incorporated into high-density lipoproteins (HDLs) [30,31,39]. The different release mechanisms are shown in Figure 1.

### 2.3. Circulating miRNAS

Once extracellular miRNAs enter the bloodstream, they circulate systemically and can be detected in plasma and other biofluids. Beyond their roles in regulating gene expression within tumor cells, extracellular miRNAs are of interest as non-invasive biomarkers. Circulating miRNAs comprise a heterogeneous mixture derived from multiple sources, including tumor cells, blood cells, and other tissues; therefore, circulating miRNA profiles often represent a composite signal rather than a tissue-specific readout [31]. Accordingly, tumor-associated changes may manifest as either increases or decreases in circulating miRNA abundance, depending on the contribution of the tumor relative to other cellular sources, and both directions of change have been investigated for diagnostic and prognostic applications [40,41,42]. Finally, accumulating evidence suggests that tumor-derived extracellular miRNAs, particularly when delivered via extracellular vesicles, can modulate the tumor microenvironment and influence distant tissues, potentially contributing to pre-metastatic niche formation [31,43]. This review focuses on circulating miRNAs as biomarkers, while noting that release mechanisms and carrier context shape detectability and functional signaling.

## 3. MiRNA in Breast Cancer

In humans, over 2600 individual mature miRNA sequences have been annotated to date. For many of them, validated targets and mechanisms have been reported, and their involvement in breast cancer progression and malignancy has been identified. For the purposes of this review, breast cancer–relevant miRNAs are grouped into four functional categories: (1) epithelial-to-mesenchymal transition (EMT) and metastasis regulators; (2) DNA damage and cell cycle control; (3) immune modulation; and (4) hormone or growth factor signaling. Some miRNAs have multiple targets and can be assigned to more than one group. This section addresses the molecular targets and the pathways regulated by the different miRNAs. Circulating miRNAs as biomarkers and potential therapeutics are covered in Section 4.

### 3.1. EMT and Metastasis Regulators

**MiR-155** was identified as oncogenic miRNA early on, and this effect is exerted through pathways that cover different cellular functions. MiR-155 silences SHIP1, an inhibitor of the PI3K/AKT pathway, driving oncogenic phenotypes in experimental models [44], and it enhances TGF-β-associated epithelial plasticity and cell migration/invasion by targeting the Ras homolog family member A (RhoA) [45]. **MiR-21** promotes invasion and therapy resistance by suppressing Phosphatase and Tensin Homolog (PTEN) [46]. ANKRD46, a protein containing multiple ankyrin repeats which is responsible for cell integrity, migration and embryo implantation was identified as direct target of miR-21 in breast cancer [47]. Elevated circulating miR-21 has been reported in liquid-biopsy studies and meta-analyses [40,41]. **MiR-10b** drives metastasis via the HOXD10→RHOC pathway. It is elevated in breast cancer both in mice and humans [48,49]. **MiR-373** promotes invasion and metastasis in part by activation and upregulation of the HIFα-TWIST pathway through mRNA silencing of the thioredoxin-interacting protein (TXNIP) [50]. **MiR-200** is considered a master regulator in EMT regulation. It targets ZEB1 and SIP1/ZEB2, transcriptional repressors of E-cadherin [51], and its dysregulation is linked to EMT in tumorigenesis [52]. **MiR-31:** In TNBC, miR-31 acts as an invasion and metastasis inhibitor via SATB2 [53]. **MiR-205** is a tumor-suppressing miRNA that has been found to be under-expressed in breast cancer and suppresses EMT through the ZEB1/2 pathway [51]. **MiR-335** suppresses metastatic cell invasion by targeting the transcription factor SOX4 and the membrane signaling glycoprotein Tenascin C (TNC) [54]. It also functions as an activator of the BRCA1 DNA repair mechanism [55]. **MiR-126** is a strong suppressor of metastasis that is selectively downregulated in many primary tumors including breast cancer [54]. It has been shown to modulate angiogenesis via VEGF-A [56] and the AKT/mTOR pathway via PIK3R2 [57]. **MiR-206** is involved in ER signaling and is downregulated in ER-positive breast cancer [58,59] where it suppresses EMT by targeting the TGF-β pathway [60]. Other pathways for tumor suppression have also been identified: in TNBC, miR-206 inhibits cell migration by direct targeting of the actin-binding protein coronin 1 [61] and it suppresses stemness and EMT through the MKL1/IL11 axis [62]. **MiR-145** suppresses cell invasion and metastasis by silencing Mucin-1 [63]. In TNBC, silence of miR-145 is responsible for enhanced cell invasion. Recovery of miR-145 results in a loss of cell invasiveness through downregulation of Arf6 [64]. **MiR-125** is downregulated in several solid tumors. Both the miR-125a and miR-125b variants function through the ERBB2/3 pathway [65], which would put it into the growth factor regulation category. However, in breast cancer, the tumor-suppressive effect of miR-125 is mediated through the induction of apoptosis by suppressing the Mucin 1 oncoprotein [66], similar to the effect of miR-145. **MiR-139** was downregulated in all tumor tissue samples compared with control tissues, and stronger downregulation correlated with higher-grade tumors and worse outcomes [67]. The mechanism found for miR-139 in breast cancer was the induction of cell cycle arrest in S phase by targeting Notch1 [68] and suppression of EMT by the silencing of CXCR4 [69]. **MiR-365** overexpression in breast cancer reduces cell growth and resistance to 5-Fluorouracil chemotherapy through GALNT4 [70], an enzyme that initiates mucin-type post-translational modification [71]. By inhibiting FOXK1, miR-365 suppresses cell growth and EMT [72]. In TNBC, miR-365 is consistently downregulated and this downregulation correlates with the metalloprotease ADAM10, which is involved in cell migration and invasion [73]. **MiR-103** regulates TNBC migration and invasion by targeting olfactomedin 4 (OLFM4) [74]. MiR-103 can promote metastasis/EMT in part by targeting DICER, attenuating miRNA biogenesis [75]. The four different variants of **miR-181** (a, b, c, d) share the identical 5’seed sequence, which is critical for recognition and binding of the target mRNA. The miR-181a, miR-181b and miR-181c variants have known roles in breast cancer [76,77]. MiR-181a is induced by TGF-β and promotes metastasis of breast cancer, which is also reflected in dramatic upregulation in metastatic breast cancer [78]. MiR-181c has an inverse, tumor-suppressive effect in TNBC [79]. The target that was identified for miR-181c is MAP4K4 [79]. **MiR-24** is up-regulated in breast cancer tissues, and high levels of miR-24 are a strong prognostic factor for the development of metastases [80,81]. The functional targets of miR-24 include enhanced hypoxia response and apoptosis resistance [80] as well as the histone variant H2AX that is involved in initiation of DNA damage response [82]. While functioning as an oncogene for other breast cancer subtypes, in TNBC, miR-24 has tumor-suppressive effects, inhibiting cell survival, EMT and tumor growth [83]. This is another example that highlights the difference between TNBC and other types of breast cancer. **MiR-425** shares a gene locus with miR-191 and it was found to be downregulated in TNBC, where it can suppress EMT through the TGF-β1/SMAD pathway [84]. In other breast cancer subtypes, miR-425 is upregulated compared to normal breast tissue [85]. The functional effects of high miR-425 levels are increased cell proliferation, accelerated cell cycle progression and enhanced PI3K/AKT pathway activation [85,86] as well as suppression of DICER translation which reduces the tumor-suppressive effect of the let-7 family miRNAs [86]. **MiR-1** is consistently downregulated in cancer and higher levels of miR-1 correlate with better therapy response [87]. Specifically in breast cancer, miR-1 has been reported to suppress cancer stem cell (CSC) stemness, proliferation and migration by inhibiting the Wnt/β-catenin signaling [88].

### 3.2. DNA Damage and Cell Cycle Control

**MiR-155** exerts oncogenic effects at the cell cycle control level. It enhances cell proliferation and rewires cellular metabolism by inhibiting FOXO3a within the PIK3R1/FOXO3a/c-Myc axis [89]. The **let-7 family** miRNAs silence the oncogenes RAS and HMGA2. Low levels of let-7 are correlated with cancer stemness and with worse outcomes in multiple tumor types including breast cancer [90]. MYCN is a direct target of **miR-34a** [91]. MiR-34a transcription is regulated by p53, and oncogenic downstream targets have been reported, including MYC-family members. High levels of miR-34a in breast cancer correlate with better outcomes and a lower risk of metastasis [92]. **MiR-335** activates the BRCA1 DNA repair mechanism [55] in addition to its previously described EMT regulation through the transcription factor SOX4 [54]. **MiR-15/16/107/195:** These four miRNAs share the same seed sequence, which means that their targets are mostly overlapping [93,94,95]. MiR-15/16 have been reported to promote apoptosis by suppressing the anti-apoptotic BCL2 protein [93] and can enhance radiosensitivity by targeting G2 checkpoint control in breast cancer [96]. **MiR-139** induces cell cycle arrest in S phase by targeting Notch1 [68] and has been identified as an EMT suppressor through the silencing of CXCR4 in breast cancer [69]. **MiR-425** shares a gene locus with miR-191, and the main effect is the suppression of DICER translation, which results in a general reduction in mature miRNA synthesis and attenuates let-7 family tumor-suppressive activity [86], enhancing the PI3K/AKT pathway signaling [85,86]. The **miR-103/107** paralogs also suppress DICER, similar to the effects described for miR-425, resulting in enhanced EMT and metastatic traits through impaired miRNA biogenesis [75]. **MiR-192** is downregulated in breast cancer compared to the adjacent healthy breast tissue, and in breast cancer cell lines, the same inverse correlation of expression with aggressive malignant phenotypes was observed [97]. The identified targets for miR-192 are Caveolin1 [97] and Rho GTPase Activating Protein ARHGAP19 [98]. The **miR-181a** and **miR-181b** variants have a oncogenic effect by silencing the expression of the ATM kinase, an early sensor for DNA damage and initiator of DNA damage repair [99]. **MiR-24** modulates DNA damage response and apoptosis by regulating H2AFX/H2AX and BCL2 [82]. As a tumor-suppressing miRNA, **miR-1287-5p** has been found significantly downregulated in TNBC, and the low expression is associated with poor prognosis. Its molecular target is the beta catalytic subunit of phosphoinositide 3-kinase (PIK3CB), and miR-1287-5p inhibits malignant growth and tumor formation while sensitizing cells to PI3K inhibitors [100].

### 3.3. Immune Modulation and Tumor Microenvironment

**MiR-155** is associated with aggressive disease. miR-155 can contribute to tumor progression through modulation of immune and inflammatory signaling [44,101,102]. By silencing the Suppressor of Cytokine Signaling 1 (SOCS1), miR-155 promotes pro-inflammatory cytokine signaling and an inflammatory tumor microenvironment. In addition, miR-155 has context-dependent roles in antitumor immunity, as demonstrated by genetic interactions with other immune-regulatory miRNAs during T cell-mediated responses [101,102]. **MiR-126** modulates the tumor immune response by repressing recruitment of mesenchymal stem cells and inflammatory monocytes [103]. High **miR-143** levels in ER-positive breast cancer tumors have been found to create a tumor microenvironment that is favorable to immune infiltration [104]. Among the miR-181 family, **miR-181a** has been implicated in immune modulation in breast cancer. Breast-cancer-derived exosomal miR-181a promotes the development of early-stage myeloid-derived suppressor cells (eMDSCs) [105]. In TNBC, miR-181a inhibits STING, a central driver of innate interferon response and immune cell activation [106].

### 3.4. Hormone and Growth Factor Signaling

**MiR-221/222** functions as a modulator of the ER pathway. It is enriched in ER-negative tumors, and its mechanism includes tamoxifen resistance along with the ER downregulation [107,108]. **MiR-133** is traditionally associated with myocardial infarction [109]; however, effects were also reported in breast cancer. Breast cancer tissues and cell lines present reduced miR-133 expression compared to healthy tissue. Delivery of miR-133 suppressed breast cancer proliferation *in vitro* and *in vivo* in a mouse model by suppressing the expression of EGFR and the phosphorylation of AKT [110]. In non-TNBC, **miR-31** is differentially expressed and its levels correlate with ER/PR status [111]. **MiR-125** was reported to be downregulated in several solid tumors. Both the miR-125a and miR-125b variants function through the ERBB2/3 pathway [65]. In breast cancer, the tumor-suppressive effect of miR-125 is mediated through the suppression of the Mucin 1 oncoprotein [66]. Expression of **miR-191** is upregulated in breast cancer compared to healthy tissue [112]. MiR-191 is a direct target of estrogen receptor activation. It enhances breast cancer progression and migration through SATB1 [113]. **MiR-382** was found to be elevated in sera of breast cancer patients compared to healthy patients [114]. The mechanism that was found for this observed correlation with increased breast cancer progression is the silencing of the Ras-related and estrogen-regulated growth inhibitor RERG [115]. This leads to increased proliferation through activation of the Ras kinase pathways. **MiR-17-92** is a cluster of miRNAs that are derived from the same locus and transcribed together. It contains miR-17, miR-18a, miR-19a, miR-20a, miR-19b-1 and miR-92a-1 [116]. While the function of miR-17-92 in breast cancer remains not fully defined and appears subtype dependent, the expression and effects observed were different between TNBC and ER-positive breast cancer. The expression of miR-17-92 is elevated in TNBC but reduced in ER-positive breast cancer. Increased expression of miR-17-92 cluster miRNAs is associated with poor outcomes in TNBC, and good outcomes in ER-positive breast cancer [116]. **MiR-143** is generally downregulated in breast cancer compared to healthy breast tissue and more aggressive or higher-grade tumors showed increasingly lower levels of miR-143 [117]. It suppresses cell proliferation through ERBB3 [118] and reduces breast cancer proliferation and stemness by targeting CD44+ [119].

In summary, the targets of the oncogenic (Table 1) and tumor-suppressive (Table 2) miRNAs can be roughly grouped into general pathways by which they effectuate their regulatory effects. Oncogenic miRNAs enhance tumor cell survival, invasion, and immune evasion by silencing tumor suppressor genes and negative regulators of growth pathways. For example, miR-425 and miR-103/107 suppress DICER [75,86], globally reducing mature miRNA biogenesis and thereby promoting oncogenic programs. Examples of enhancers of EMT promoting tumor invasion and metastasis are found in miR-21, miR-10b, and miR-373 [46,48,50]. A third group of oncogenic miRNAs operates within hormone-response pathways; for example, miR-191 is an estrogen-responsive oncogenic miRNA in breast cancer [113]. MiR-155 is more strongly supported in this section as an immune/inflammation-associated miRNA [101,102]. Tumor-suppressive miRNAs counteract malignant progression by strengthening key regulatory programs. They can enforce cell-cycle control, promote apoptosis, limit invasion by reducing expression of pro-invasive proteases, and help maintain epithelial identity by antagonizing EMT. For example, the let-7 family suppresses oncogenic drivers such as RAS and HMGA2 [90], and miR-34a targets MYCN as part of the p53 tumor-suppressor network [91]. MiR-205 and miR-200 maintain epithelial integrity by abolishing transcriptional repression of E-cadherin [51,52]. miR-126, miR-145, and miR-125 suppress invasion and tumor-promoting phenotype [56,57,64,65]. Other miRNAs such as miR-1and miR-133, target pathways that drive cell cycle progression such as Wnt/β-catenin, EGFR/AKT, and growth/survival signaling [88,110]. For five miRNAs, the miR-17-92 cluster, miR-425, miR-181, miR-31 and miR-24, differential effects between TNBC and ER-positive breast cancer were reported (Table 3).

## 4. Circulating miRNAs in Clinical Diagnostics and Therapy

### 4.1. Liquid Biopsy miRNA in Diagnostic/Therapeutic Applications and Data Meta-Analyses

Progress in medical diagnostics allows for the analysis of extracellular miRNA in liquid biopsies. Liquid biopsy samples from plasma can be obtained minimally invasively in standard blood testing and plasma miRNA signatures can serve as biomarkers for diagnosing cancer, assessing stage and metastatic progression of cancer and evaluation of prognosis and recurrence risk [19,123]. 

A recent study on a small Finnish TNBC cohort identified three differentially expressed miRNAs whose levels distinguished recurrent from non-recurrent cases: miR-21 was higher in recurrent disease, associated with worse recurrence-free survival with a hazard ratio (HR) of 1.87; miR-16 and miR-26b were associated with improved recurrence-free survival at HR = 0.53 and HR = 0.52, respectively [124]. A 2024 review of recent and ongoing studies on liquid biopsies in TNBC identified elevated miR-21 and miR-155 as markers for advanced or metastatic diseases, while miR-205 was often downregulated [125]. A multicohort study conducted with serum samples from 139 TNBC patients (35 stage-I, 43 stage-II, 31 stage-III, and 30 stage-IV) and 51 healthy controls confirmed three targets: miR-21 at an individual AUC of 86.9% and miR-155 at AUC = 87.0% (upregulated) and miR-205 with an AUC of 81.9% (downregulated) and the panel combining these three targets achieved an AUC of 96.1% [126].

The Cancer Genome Atlas (TCGA) is a database containing the genomic, epigenomic, transcriptomic, and proteomic data of large numbers of primary tumor samples across multiple cancer types. Public platforms such as *OncomiR* [127], *dbDEMC 3.0* [128], and *miRCancer* [129] narrow these datasets and published studies the miRNA level and allow for large-scale bioinformatic and meta-analytic studies. 

Meta-analyses and clinical studies have evaluated circulating miRNAs as diagnostic and prognostic biomarkers in breast cancer, including exosomal/serum miR-21 and circulating miR-155 [40,41,130,131]. 

Therapeutic modulation of miRNAs has progressed into the clinic. While not targeting breast cancer specifically, both replacement and inhibiting therapies have been evaluated in clinical studies. The miRNA mimic trials were targeting miR-34 and miR-16 and the anti-miR inhibition trials were directed towards miR-155 and miR-122 [132,133,134,135]. Candidates for application in breast cancer would be anti-miR drugs targeting miR-21, miR-155, miR-10b, and miR-103/107 for the inhibitory strategy or following the replacement strategy using mimics for the tumor-suppressive miRNAs miR-34a, or the miR-16 family. A promising option could be a combination of these novel miRNA therapies with the standard of care (chemotherapy, targeted agents, or immunotherapy) to enhance the presently available treatments and improve prognosis.

One big challenge in the implementation of therapeutic miRNAs is the delivery mechanism for miRNA mimics or anti-miRs. Naked miRNAs in circulation undergo rapid degradation by nucleases and require protective delivery vehicles or chemical modification to improve their stability. Another challenge is the penetration of cellular membranes from the circulation into the cytoplasm of the targeted cells. Another matter of concern are off-target effects: synthetic miRNA mimics and inhibitors could be toxic by inadvertently modulating unintended mRNAs and disrupting normal cellular processes. Modified oligonucleotides and synthetic delivery platforms can trigger immune activation or inflammatory responses [136,137,138]. The miRNA delivery platforms that are currently being explored are lipid nanoparticles and exosome-based systems [139,140]. Delivery of miRNAs using liposome nanoparticles has already been applied in clinical studies, such as MRX34 [132]. 

While exosomes excel at delivering biological cargoes, implementation typically requires optimization of loading and targeting strategies [140,141]. There has been promising progress using exosomes loaded with miR-205 in a mouse *in vivo* study [142], but this remains preclinical and has not yet been translated to breast cancer clinical trials.

Considering breast cancer-specific miRNA trials, there is only one ongoing trial using miRNAs to target breast cancer. The INT-1B3 study uses a lipid nanoparticle-formulated miR-193a mimic to treat solid tumors, with breast cancer patients being eligible to enroll. While the investigation design for this trial NCT04675996 has been published [143], this phase I/Ib study only assesses the safety, pharmacology and pharmacokinetics of the drug formulation and no results for the breast cancer cohort have been communicated. There have been several clinical trials in which miRNAs were investigated as biomarkers for intervention, and some have shown predictive value for treatment response (Table 4).

### 4.2. Challenges of miRNA-Based Analytics

A review on the challenges of using circulating miRNAs as cancer biomarkers highlighted the need for standardization both in analytical protocols and in selected targets. Many studies have reported recurring candidate miRNAs (e.g., miR-21); however, variability in sample collection and preparation and small cohorts limited the translation into clinical application [123]. 

MiRNA analysis is a relatively recent field, so there has been only limited clinical standardization while there are numerous variables to consider. In addition to the inherent differences between patients, the variation begins with the choice of sample and sample acquisition. Extracellular miRNA can be extracted from the whole blood either via the blood plasma or serum. Pre-analytical variables, including storage conditions and handling, can influence measured miRNA levels; therefore, consistent sample processing and avoidance of unnecessary handling steps are recommended. 

Release of miRNAs from platelet cells during coagulation can affect the miRNA levels in serum. On the other hand, the anticoagulant heparin inhibits PCR amplification and must be removed by an additional step of either RNA purification or heparinase digestion. Using EDTA or citrate as an anticoagulant avoids this issue; however, careful handling remains essential to minimize hemolysis and other pre-analytical artifacts [148,149]. 

Contamination from erythrocyte hemolysis can make samples unusable, as circulating miRNA profiles can be strongly influenced by blood-cell-derived miRNAs [150,151] and even low levels of hemolysis can substantially alter measured miRNA concentrations [151,152]. Measuring free hemoglobin (e.g., oxyhemoglobin) in samples by spectrophotometry can ensure the absence of hemolysis [149,152]. 

The choice of anticoagulant and whether to use any at all must be standardized. MiRNA extraction can be performed with column-based systems or by TRIzol. Measurement of isolated miRNA is done by qRT-PCR, miRNA-Seq or NanoString hybridization. New normalization strategies have to be implemented due to a lack of housekeeping miRNAs and even within one method there are different platforms such as qRT-PCR and ddPCR. All these considerations have been described and discussed repeatedly [148,149,150,153,154,155,156], but standardization remains limited and inconsistently adopted across studies [123,149]. A consistent protocol with standardized steps could greatly improve reproducibility and consistency between research groups and studies. One suitable example could comprise blood samples using EDTA as an anticoagulant, plasma separation by centrifugation with defined RCF and time, spectrophotometry of plasma to exclude hemolysis, then column-based RNA extraction followed by miRNA-seq.

## 5. Discussion

MiRNAs play a significant role in breast cancer biology. Their production and secretion are dysregulated in tumors and can affect the direct tumor microenvironment but also reach the circulation and have systemic effects. After being secreted in extracellular vesicles or bound to proteins, these circulating miRNAs in some contexts modulate immune responses and influence distant tissues, and have been implicated in pre-metastatic niche conditioning to facilitate cancer progression, tumor invasion, and malignant cell migration [31,43]. 

The miRNAs can have oncogenic, cancer-progressing effects; for example, miR-21 and miR-10b promote invasion and metastasis [46,48], miR-155 drives immune modulation [101,102], and miR-373 is a driver of EMT via the TXNIP–HIF1α–TWIST axis [50]. On the other hand, tumor-suppressive miRNAs such as, miR-34a, miR-126, miR-145, and miR-205 restrain proliferation, angiogenesis, and stemness, and their downregulation has been associated with more aggressive disease and adverse outcomes in multiple studies [51,56,57,63,64,92,103].

An interesting observation became apparent during the compilation of the effects and mechanisms of different miRNAs in breast cancer. Several miRNAs demonstrate opposite effects in TNBC compared to ER-positive tumors. The miR-17-92 cluster correlates with poor prognosis when overexpressed in TNBC, yet is reduced and potentially protective in ER positive disease [116]. Likewise, miR-31 inhibits invasion in TNBC and shows differential expression associated with ER/PR status [53,111]. MiR-425 transcription is directly mediated through an ER-responsive cluster [157], resulting in high levels in ER- positive breast cancer. MiR-181 family members display subtype-specific duality. MiR-181a/b enhance metastasis via ATM suppression and impaired DNA damage response [99], whereas miR-181c acts as a tumor suppressor in TNBC through MAP4K4 silencing [79]. Similarly, miR-24 has arm-specific isoforms with differential effects. miR-24-3p functions as an oncogene in luminal cancer, by promoting hypoxia tolerance [80,81] and resistance to apoptosis. However, in TNBC miR-24-2-5p exerts tumor-suppressive effects by inhibiting EMT and cell survival [82,83]. 

These contrasting behaviors highlight that while miRNA targets are determined by the miRNA seed sequence, the resulting phenotype of miRNA dysregulation is dependent on cellular context and signaling state, including hormone pathway activity. Also, similar seed sequences that are regarded as subtypes of the same miRNA can show considerable variability in targeted pathways and resulting phenotypes. A deeper understanding of the interaction of different pathways and how miRNAs interact with them is important. Understanding these interactions and being able to individually characterize the malignancies of the patient is critical to be able to implement the best possible therapeutic strategies [131].

Another important aspect that has become evident is that several miRNAs regulate overlapping pathways from different cellular mechanisms such as EMT, cell cycle control, hormone and growth factor signaling and immune modulation. These miRNAs would not be suitable as therapeutic targets because of the variety of possible downstream effects.

An interesting observation is the inter-miRNA regulation found for miR-425/103/107 that target DICER. Given the biogenesis pathways described above, dysregulation at the DROSHA/DICER level will lead to reduced mature miRNA that is incorporated into the RISC complex which effectuates mRNA silencing. Overexpression of these miRNAs is generally associated with cancer progression and negative outcomes. 

The expression and function of miRNAs can differ between molecular subtypes of breast cancer: ER-negative tumors are enriched in miR-221/222, which confer tamoxifen resistance, while miR-206 is suppressed in ER-positive breast cancer but acts as a strong inhibitor of migration in TNBC [58,61]. Similarly, the miR-17-92 cluster shows divergent roles, being elevated in TNBC along with an unfavorable prognosis, but reduced and favorable in ER-positive disease [116].

One important question that is always present within the context of circulating miRNA as cancer biomarkers is the extent to which the miRNA dysregulation in the tumor tissue is reflected in the circulation. For example, some studies found that a large proportion of circulating miRNAs do not correlate with tissue-specific levels across cancer types [158]. This suggests that circulating signatures do not directly reflect tumor expression, but rather a mixture of systemic responses and selective miRNA release mechanisms. This limited reproducibility of circulating miRNA biomarkers across studies brings into question the reliability of clinical applications and highlights the necessity of careful standardization [159]. 

MiRNA-directed therapeutics are only just entering the field of clinical studies with a few candidate compounds and there are still significant hurdles to overcome. The first issue is the identification of the target miRNA or combination of miRNAs that are specific for at least a subtype of breast cancer. The next step is the reliable identification of patients that are good candidates for these new therapies, at which point the need for standardized diagnostics comes back into play. The next issue is the stable delivery of either anti-miR or miRNA mimic small oligonucleotide therapeutics to the target tissues, which requires chemical modification to stabilize the therapeutics preventing degradation and transport vehicles in the form of lipid nanoparticles or exosomes. Finally, there is the possibility of off-target effects affecting other miRNA-regulated pathways and unintended immune activation and inflammation.

## 6. Conclusions

Circulating miRNAs could present a translational opportunity for minimally invasive biomarkers for diagnosis, prognosis, and therapy guidance. Panels combining the different oncogenic and tumor-suppressive miRNAs could be used to establish personalized treatments. The specific miRNA levels for the different breast cancer subtypes can also be used to help direct treatment, such as ER-related miRNAs (miR-206, miR-221/222) or TNBC-associated regulators (miR-31, miR-365).

In conclusion, breast cancer-associated miRNAs extend beyond local tumor control to influence systemic disease progression through their presence in circulation. Their dysregulation across subtypes reflects the biological heterogeneity of breast cancer and underscores the potential of miRNA-based biomarker panels to augment current diagnostic and therapeutic frameworks. The integration of circulating miRNA signatures into clinical practice could provide earlier detection, more accurate prognosis, and new avenues for targeted intervention in breast cancer.

## Figures and Tables

**Table 1:** Oncogenic miRNAs in breast cancer with molecular targets and effect on prognosis.

miRNA	Target	Effect on Breast Cancer Prognosis	Reference
miR-21	PTEN	Promotes invasion and metastasis; associated with poor prognosis; elevated also in circulation	[40,41,46]
miR-155	Multiple targets in EMT-proliferation and immune/inflammatory pathways	Associated with aggressive disease; promotes inflammation and immune modulation	[44,45,89,101,102,120]
miR-10b	HOXD10 → RHOC pathway	Driver of metastasis; elevated in tumors; poor prognosis	[48]
miR-221/222	Estrogen receptor (ER) pathway	Enriched in ER-negative tumors; induces tamoxifen resistance; poor prognosis	[107,108]
miR-373	TXNIP (thioredoxin-interacting protein)	Promotes EMT, invasion, and metastasis via HIFα-TWIST activation	[50]
miR-191	SATB1; estrogen receptor-responsive	Enhances tumor progression and migration; high levels → poor prognosis	[113]
miR-382	RERG (Ras-related and estrogen-regulated growth inhibitor)	Promote proliferation and tumor initiation; serum levels correlate with disease	[114,115]
miR-103/107	OLFM4; DICER	Promotes migration/invasion in TNBC (OLFM4) and metastasis/EMT via DICER suppression	[74,75]

**Table 2:** Tumor-suppressive miRNAs in breast cancer with molecular targets and effect on prognosis.

miRNA	Target	Effect on Breast Cancer Prognosis	Reference
miR-200	ZEB1/2	Suppresses EMT; low expression → poor prognosis	[51,52]
let-7 family	RAS, HMGA2	Low levels → stemness and poor outcome	[90]
miR-34a	MYC, p53-regulated	High expression → better prognosis	[91,92]
miR-31	SATB2	Inhibits invasion/metastasis in TNBC; elevated in non-TNBC (correlates with ER/PR status)	[53,111]
miR-205	HER3, VEGF-A, ZEB1/2	Suppresses EMT; under-expressed → poor prognosis	[51,121,122]
miR-335	SOX4, Tenascin C, BRCA1	Suppresses invasion; activates DNA repair; better outcome	[54,55]
miR-126	VEGF-A, PIK3R2	Suppresses metastasis and angiogenesis; low levels → poor prognosis	[54,56,57,103]
miR-206	TGF-β, Coronin 1, MKL1/IL11	Downregulated in ER-positive BC; high levels → reduced EMT and stemness	[58,59,60,61,62]
miR-1	Wnt/β-catenin	Downregulated in BC; high levels → better therapy response	[87,88]
miR-133	EGFR, AKT	Downregulated; restoration suppresses proliferation	[110]
miR-125a/b	ERBB2/3, MUC1	Induces apoptosis; low expression → poor prognosis	[65,66]
miR-145	MUC1, Arf6, ERBB3	Suppresses invasion/metastasis; low expression in BCs; poor outcome in TNBC	[63,64,118]
miR-139	Notch1, CXCR4	Suppresses EMT; low levels → high-grade tumors, poor outcome	[67,68,69]
miR-143	ERBB3, CD44+	Reduces proliferation/stemness; high levels → favorable immune infiltration	[104,117,118,119]
miR-365	GALNT4, FOXK1, ADAM10	Downregulated in TNBC; high levels → better prognosis and chemosensitivity	[70,72,73]
miR-192	Caveolin1, RHGAP19	Downregulated; low expression → poor prognosis	[97,98]
miR-1287	PI3K β subunit	Downregulated; low levels → poor prognosis	[100]

**Table 3:** MiRNAs in breast cancer exhibit subtype-specific effects.

miRNA	TNBC	Other Breast Cancer Subtypes	Reference
miR-17-92 cluster	Levels elevated, poor prognosis	Reduced levels in ER-positive breast cancer, positive prognosis	[116]
miR-425	Downregulated in TNBC, suppresses EMT through the TGF-β1/SMAD pathway	Upregulated in non-TNBC, DICER inhibition and PI3K/AKT activation enhance proliferation, poor prognosis	[84,85,86]
miR-181	miR-181c variant is tumor-suppressive in TNBC through MAP4K4	Promotes metastasis, inhibits DNA damage repair, inhibits immune response, poor outcomes	[76,77,78,79,99,105,106]
miR-31	Inhibits invasion/metastasis in TNBC through SATB2	Overexpressed in non-TNBC (associated with ER/PR status)	[53,111]
miR-24	Inhibits cell survival, EMT and tumor growth in TNBC	Oncogenic in most breast cancer, promotes metastasis and apoptosis resistance	[80,81,82,83]

**Table 4:** Clinical trials investigating miRNAs during breast cancer therapy.

Study	Context	miRNAs	Patients	Source	Method	Outcome Summary
CTRIAL-IE (ICORG10/11) 2020 [144]	NACT response prediction	let-7a, miR-21, miR-145, miR-155, miR-195	114	Whole blood	qRT-PCR	Low baseline miR-21 (OR 0.539, *p* < 0.05) and miR-195 (OR 0.561, *p* < 0.1) correlate with better chemotherapy response
NCT01722851, 2022 [145]	NACT response prediction	miR-145	120	Whole blood	qRT-PCR	Increase in miR-145 at therapy midpoint predicts RFS (HR 0.59, *p* = 0.054) and DFS (HR 0.57, *p* = 0.05)
miR-1 NAC biomarker, 2024 [146]	NACT response prediction	miR-1	80	Serum	qRT-PCR	High baseline miR-1 correlates with DSF after NACT (HR = 0.216, *p* = 0.033)
ICORG10/11 NAC toxicity 2023 [147]	NACT toxicity study	miR-195, miR-10b, miR-145, miR-21, miR-155	101	Whole blood	qRT-PCR	Low miR-195 predicted neutropenia, high h miR-10b predicted anemia, high h miR-145 predicted nausea, low miR-21 predicted mucositis

## Data Availability

No new data was generated in this review.

## Abbreviations

The following abbreviations are used in this manuscript:

ADAM10	A Disintegrin and Metalloproteinase Domain-Containing Protein 10
AGO	Argonaute Protein
AKT	Protein Kinase B
Arf6	ADP-Ribosylation Factor 6
ARHGAP19	Rho GTPase-Activating Protein 19
BCL2	B-Cell Lymphoma 2
BRCA1	Breast Cancer Type 1 Susceptibility Protein
CD44	Cluster of Differentiation 44
MYC(c-Myc)	MYC proto-oncogene protein
CSC	Cancer Stem Cells
ctDNA	Circulating Tumor DNA
CXCR4	C-X-C Motif Chemokine Receptor 4
ddPCR	Droplet Digital PCR
DFS	Disease-Free Survival
DGCR8	DiGeorge Syndrome Critical Region 8 microprocessor complex subunit
EDTA	Ethylenediaminetetraacetic Acid
EGFR	Epidermal Growth Factor Receptor
eMDSC	Early Stage Myeloid-derived Suppressor Cell
EMT	Epithelial-to-Mesenchymal Transition
ER	Estrogen Receptor
ERBB	Erbb Receptor Tyrosine Kinase Family
FOXK1	Forkhead Box Protein K1
FOXO3a	Forkhead Box Protein O3a
GALNT4	Polypeptide N-Acetylgalactosaminyltransferase 4
H2AFX/H2AX	H2A Histone Family Member X
HDL	High-density Lipoprotein
HER2	Human Epidermal Growth Factor Receptor 2
HIFα	Hypoxia-Inducible Factor α Subunit
HMGA2	High-Mobility Group AT-Hook 2
hnRPA2B1	Heterogeneous Nuclear Ribonucleoprotein A2/B1
HOXD10	Homeobox D10
ICIs	Immune Checkpoint Inhibitors
IL11	Interleukin 11
isomiR	microRNA isoform
MAP4K4	Mitogen-Activated Protein Kinase Kinase Kinase Kinase 4
miRNA	microRNA
MKL1	Myocardin-like Protein 1
mRNA	messenger RNA
mTOR	Mechanistic Target of Rapamycin
MUC1	Mucin 1
MYCN	N-Myc Proto-Oncogene Protein
NACT	Neoadjuvant Chemotherapy
OLFM4	Olfactomedin 4
oncomiR	Oncogenic microRNA
PI3K	Phosphoinositide 3-kinase
PIK3R	PI3K Regulatory Subunit
PR	Progesterone Receptor
Pre-miRNA	Precursor microRNA
Pri-miRNA	Primary microRNA
PTEN	Phosphatase and Tensin Homolog
RAS	RAS proto-oncogene family (Rat sarcoma virus oncogene homolog)
RCF	Relative Centrifugal Force
RERG	Ras-Related and Estrogen-Regulated Growth Inhibitor
RFS	Relapse-Free Survival or Relapse-Free Survival
RhoA	Ras Homolog Family Member A
RHOC	Ras Homolog Family Member C
RISC	RNA-Induced Silencing Complex
SATB	Special AT-rich Sequence-binding Protein
SHIP1	SH2-Containing Inositol Polyphosphate 5-Phosphatase 1
SIP1	Smad-Interacting Protein 1
SMAD	SMAD family (Sma and Mad homologs)
SOCS1	Suppressor of Cytokine Signaling 1
SOX4	SRY-Box Transcription Factor 4
STING	Stimulator of Interferon Genes
SYNCRIP	Synaptotagmin Binding Cytoplasmic RNA Interacting Protein
TGF-β	Transforming Growth Factor Beta
TNBC	Triple-Negative Breast Cancer
TNC	Tenascin C
TXNIP	Thioredoxin-Interacting Protein
UTR	Untranslated Region
VEGF-A	Vascular Endothelial Growth Factor A
YBX1	Y Box Binding Protein 1
ZEB	Zinc Finger E-Box Binding Homeobox
